# Mechanisms Underlying Motivational Dysfunction in Schizophrenia

**DOI:** 10.3389/fnbeh.2021.709753

**Published:** 2021-09-10

**Authors:** Youssuf Saleh, Isaac Jarratt-Barnham, Emilio Fernandez-Egea, Masud Husain

**Affiliations:** ^1^Nuffield Department of Clinical Neurosciences, John Radcliffe Hospital, University of Oxford, Oxford, United Kingdom; ^2^Department of Psychiatry, Herchel Smith Building for Brain & Mind Sciences, University of Cambridge, Cambridge, United Kingdom; ^3^Cambridge Psychosis Centre, Cambridgeshire and Peterborough NHS Foundation Trust, Cambridge, United Kingdom

**Keywords:** effort-based decision making, motivated action selection, apathy, schizophrenia, negative symptom, reward based learning

## Abstract

Negative symptoms are a debilitating feature of schizophrenia which are often resistant to pharmacological intervention. The mechanisms underlying them remain poorly understood, and diagnostic methods rely on phenotyping through validated questionnaires. Deeper endo-phenotyping is likely to be necessary in order to improve current understanding. In the last decade, valuable behavioural insights have been gained through the use of effort-based decision making (EBDM) tasks. These have highlighted impairments in reward-related processing in schizophrenia, particularly associated with negative symptom severity. Neuroimaging investigations have related these changes to dysfunction within specific brain networks including the ventral striatum (VS) and frontal brain regions. Here, we review the behavioural and neural evidence associated with negative symptoms, shedding light on potential underlying mechanisms and future therapeutic possibilities. Findings in the literature suggest that schizophrenia is characterised by impaired reward based learning and action selection, despite preserved hedonic responses. Associations between amotivation and reward-processing deficits have not always been clear, and may be mediated by factors including cognitive dysfunction or dysfunctional or self-defeatist beliefs. Successful endo-phenotyping of negative symptoms as a function of objective behavioural and neural measurements is crucial in advancing our understanding of this complex syndrome. Additionally, transdiagnostic research–leveraging findings from other brain disorders, including neurological ones–can shed valuable light on the possible common origins of motivation disorders across diseases and has important implications for future treatment development.

## Introduction

Negative symptoms are a core feature of schizophrenia, observed in up to 90% of patients at first-episode psychosis (Kraepelin, 1921; Bleuler, 1950; Mäkinen et al., 2008; An der Heiden et al., 2016). They include a constellation of symptoms unified by the loss of “vital properties” such as speech, motivation and self-expression (Berrios, 1985). Despite being less prominent in presentation than the *positive/psychotic* features of schizophrenia (e.g., hallucinations and delusions), negative symptoms are independently associated with poor functional outcomes (Milev et al., 2005; Rabinowitz et al., 2012, 2013; Fervaha et al., 2014) and subjective well-being (Strauss et al., 2012), and can predict future psychotic episodes (Piskulic et al., 2012). To date, there are no licensed treatments specifically targeting negative symptoms, in part likely due to a lack of understanding of their underlying mechanisms. A better characterisation of such mechanisms might therefore play a key role in treating their debilitating effects across psychiatric conditions.

Recent development of behavioural paradigms to investigate negative symptoms has significantly improved our understanding of their phenotype (Gold et al., 2015; Culbreth et al., 2018). Importantly, these behavioural measures have been related directly to specific neural regions and networks in the brain (Chase et al., 2018; Culbreth et al., 2018). Here we discuss the history and current mechanistic understanding of motivational aspects of the negative syndrome considering these developments, whilst highlighting current gaps and future therapeutic possibilities.

### Negative Symptoms: The State of Play

Our conceptual understanding has progressed considerably since the formalisation of a “negative syndrome” as early as 1974 (Strauss et al., 1974). Current classification of the negative syndrome includes five symptom domains as agreed upon by the National Institute of Mental Health (NIMH) consensus statement (Kirkpatrick et al., 2006). These are: alogia, anhedonia, avolition, asociality, and blunted affect (see Table 1 for definitions). Questionnaire measures, which can help to support clinical diagnosis, have been adapted to reflect the evolving definition of the negative syndrome (Kirkpatrick et al., 2011).

**TABLE 1:** Definitions of negative syndrome domains as defined by NIMH consensus.

NIMH domain	Definitions based on Kirkpatrick et al. (2011)
Anhedonia	Reduced intensity and frequency of current or future pleasurable activities.
Avolition	A reduction in the initiation of and persistence in activity in relation to internally guided behaviour and experiences.
Asociality	Reduced social activity accompanied by a decreased interest in forming close relationships with others.
Alogia	A reduction in speech quantity, and spontaneous elaboration in conversation.
Blunted affect	A decrease in outward expression of emotion including facial, vocal and expressive gestures.

**FIGURE 1:**
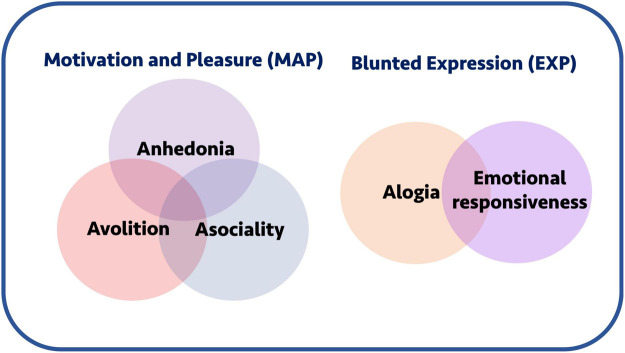
Negative symptoms and conceptual groupings. Negative symptoms are often conceptually grouped into a motivation and pleasure (MAP) and blunted expression (EXP) components. The former includes anhedonia, avolition, and asociality, whereas the latter includes alogia and emotional responsiveness. Collectively, the motivation-pleasure symptoms are also sometimes referred to using the umbrella term “apathy.”

Factor analyses of these questionnaire data have shed further light on possible underlying structures of the negative syndrome. For example, the results of some studies suggest that negative symptom domains aggregate into a two-factor structure where alogia and blunted affect form a “Diminished Expression” (EXP) factor, while the remaining domains form a “Motivation and Pleasure” (MAP) factor, which can be referred to as “apathy” (Figure 1; Kimhy et al., 2006; Nakaya and Ohmori, 2008; Foussias and Remington, 2010; Horan et al., 2011; Kirkpatrick et al., 2011; Kring et al., 2013). This dichotomised view of negative symptoms is reflected in the most recent version of the DSM-5 (Tandon et al., 2013). However, other findings suggest a more complex construct, providing some evidence against a simplistic two factor model of negative symptoms (Strauss et al., 2018, 2019a,b).

Some challenges with frameworks based on questionnaire analyses may be partly due to the use of different instruments to assess negative symptoms (Garcia-Portilla et al., 2015; Kumari et al., 2017). Previous studies have relied on questionnaires containing symptoms that are no longer considered part of the consensus statement definition of the negative syndrome (Peralta and Cuesta, 1995; Sayers et al., 1996; Kumari et al., 2017). For example, the widely used PANSS questionnaire (Positive And Negative Syndrome Scale) includes “abstract thinking” as a negative symptom, whereas the SANS (Scale for the Assessment of Negative Symptoms) questionnaire includes an “inattention” domain (Andreasen et al., 1991; Mortimer, 2007). Both these components may reflect general cognitive dysfunction, currently considered to arise separate from negative symptom mechanisms in schizophrenia (Kirkpatrick et al., 2006).

It is evident that the complex negative syndrome remains to be fully understood. While questionnaire analyses may advance our understanding at the structural level, they lack the ability to provide deeper mechanistic insight at behavioural and anatomical levels. One potential way to address the current knowledge gap is by application of an endo-phenotyping approach to advance a neurocognitive framework of this syndrome. A key area where this is being applied is in disorders of motivation.

### Effort-Based Decision Making Framework for Motivation Deficits

**FIGURE 2:**
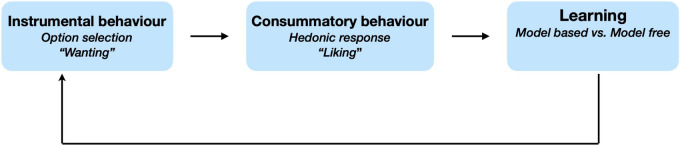
Phases of effort-based decision making for rewards. Effort based decisions making can occur across three conceptual phases. Option selection includes the processes of weighing up components of decisions and behaviourally manifests as wanting. Consummatory phase refers to the hedonic response and represents the experience of liking. Learning reflects the process of updating beliefs based on a representation of the environment that is structured (i.e., model based) or otherwise (model free). Framework adapted from Berridge and Robinson (2003) and Husain and Roiser (2018).

Motivation deficits appear to be central to the negative syndrome (Strauss et al., 2019c, 2021). An emerging framework that has been used to conceptualize the motivational aspects of negative symptoms is that of effort-based decision making for rewards (EBDM). Paradigms that assess EBDM, some translated from animal studies, have the potential to provide important insights into the phenotypes of neuropsychiatric disease by probing different phases of behaviour (Husain and Roiser, 2018). These include the instrumental, consummatory and learning phases of motivated behaviour outlined in Figure 2 and explained below.

When deciding whether to pursue a course action, an individual may weigh up its potential benefits (e.g., reward/avoiding punishment) against its costs (e.g., effort/risk) vs. other possible actions–a process referred to as “option selection” (Husain and Roiser, 2018). Alternatively, having invested some effort towards a specific goal, one may re-evaluate the merit of sustaining further effort in its pursuit. Paradigms examining these processes in patient groups can manipulate the benefits and costs of actions systematically to highlight potential deficits in effort-based decisions (Treadway et al., 2009; Le Heron et al., 2018b). Some frameworks conceptualise these processes as encompassing the “wanting” or instrumental components of motivated behaviour to seek a particular outcome (Salamone and Correa, 2012; Thomsen et al., 2015).

Whereas the decision making process represents the “wanting” aspect of behaviour, consummatory behaviour encompasses the hedonic response or the “liking” component when interacting with the goal of behaviour (Salamone and Correa, 2012; Thomsen et al., 2015). When an individual engages in pleasurable activity (e.g., watching a comedy), they may respond explicitly (e.g., rating their experience), or implicitly (e.g., smiling) (Berenbaum and Oltmanns, 1992; Berridge and Robinson, 2003). One or more of these responses may be altered in patients with negative symptoms (Berenbaum and Oltmanns, 1992; Cohen and Minor, 2010).

Having engaged in a behaviour with an unexpected positive/negative outcome, expectations of reward may be updated in relation to behaviour. In laboratory settings, this can be achieved by varying the frequency of rewarding different actions. For example, one response may be rewarded 90% of time and another only 60% of the time (Hernaus et al., 2018; Culbreth et al., 2021). Over the course of an experiment, participants learn to favour one action based on the frequency of reward (or reinforcement schedule). Deficits in the learning process may impair individuals from representing the value of reward appropriately to guide future motivated behaviour to influence future decisions.

The last decade has seen a significant rise in EBDM studies in schizophrenia spanning several of these components of decision making (Gold et al., 2013; Barch et al., 2014; Albrecht et al., 2019; Chang et al., 2019, 2020; Cooper et al., 2019; Culbreth et al., 2021). The investigations that have been performed aimed to probe cognitive mechanisms underlying behavioural deficits, including negative symptoms. Phenotypes can be captured using tasks that probe different aspects of EBDM based on behavioural performance, but they can also be derived from computational modelling of behaviour on such tasks and subsequently correlated with symptoms of interest. This approach has been used to investigate apathy and anhedonia across several disorders (Muhammed et al., 2016; Husain and Roiser, 2018; Le Heron et al., 2018a,b; Pessiglione et al., 2018; Berwian et al., 2020). As both these symptoms occur in the negative syndrome, it is possible that analysis of performance on EBDM tasks can also add value to the mechanistic understanding of negative symptoms in schizophrenia.

### Instrumental Behaviour: Option Selection

**FIGURE 3:**
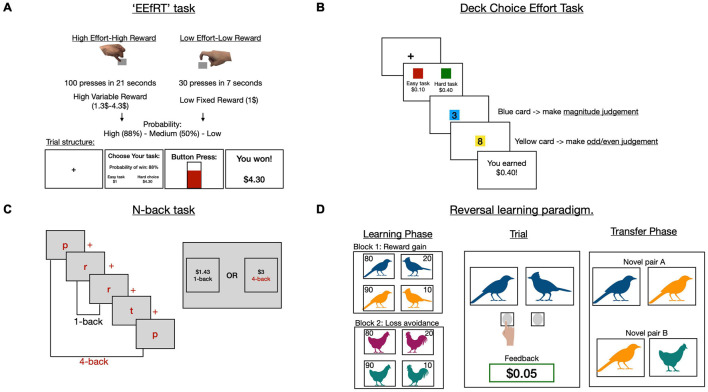
Effort based decision making tasks in schizophrenia. Schematic representation of four paradigms **(A–D)**. The EEfRT task **(A)** varies physical effort in return for monetary reward. Participants choose between a high effort-high reward condition and low effort-low reward condition. Additionally, probability of winning varies across trial types. Adapted from Treadway et al. (2009). The deck choice effort task and the N-back task **(B,C)** both use cognitive instead of physical effort in a similar task structure to the EEfRT task, although probability is not varied. **(B)** In the deck choice task, high effort involves serially alternating between two mental activities, whereas only one mental activity is performed in the low effort option. **(C)** In the N-back task participants are serially presented with a list of letters and asked if the same letter was presented N steps back. They choose between a 1-back trial for low reward or a higher N-back (e.g., 4-back) for higher reward. **(D)** Learning paradigms involve choosing between two partially reinforced images to either gain reward, or avoid loss (see subsection “Reinforcement learning of reward value”). In a learning phase, only two images are presented per trial and one type of outcome is varied per block (reward gain vs. loss avoidance). A subsequent transfer phase uses novel combinations of visual stimuli to investigate learning behaviour across trial types.

One of the earliest and most commonly used EBDM paradigms is that developed by Treadway et al. (2009, 2015) known as the “Effort Expenditure for Rewards Task” (EEfRT). This paradigm and its variations have been widely used to probe option selection in patients and healthy controls (Fervaha et al., 2013; Barch et al., 2014; Hartmann et al., 2015; Treadway et al., 2015; Chang et al., 2019) and have demonstrated good test-retest reliability (Reddy et al., 2015) and external validity (Horan et al., 2015). In this task participants exert physical effort either by selecting to press on a key using the dominant index finger (low-effort) or the non-dominant little finger (high-effort). While the low effort condition is paired with a single fixed low reward (e.g., $1), the high effort condition can be paired with one of four possible higher reward magnitudes (e.g., $1.3–$4.3). Additionally, the outcome probability is varied across all conditions to incorporate an element of uncertainty (25, 50, or 88%). On a trial by trial basis, participants are offered either a low reward–low effort condition or a high reward–high effort condition with outcome probability varied (Figure 3A).

The most consistent finding reported using this task is that schizophrenia patients paradoxically accept less offers than healthy controls when reward levels and probabilities are the *highest* (Fervaha et al., 2013; Gold et al., 2013; Barch et al., 2014; Hartmann et al., 2015; Horan et al., 2015; Reddy et al., 2015; Treadway et al., 2015; McCarthy et al., 2016; Moran et al., 2017). Further, some studies demonstrate that this effect is most pronounced in patients with higher negative symptom severity (Gold et al., 2013; Hartmann et al., 2015; Cooper et al., 2019), although this finding is not consistent (Fervaha et al., 2013; Barch et al., 2014; Horan et al., 2015; Moran et al., 2017). These inconsistencies might arise due to a variety of reasons which include use of different analysis approaches (Fervaha et al., 2013; Horan et al., 2015), questionnaire types (Fervaha et al., 2013; Hartmann et al., 2015; Moran et al., 2017) or task variations (Horan et al., 2015). In those investigations where there were significant associations between performance and negative symptoms, correlations tended to bias toward the MAP components (Figure 1) of the negative syndrome (i.e., avolition, asociality, and anhedonia) and not the expressive component (i.e., alogia and blunted affect) (Fervaha et al., 2013; Barch et al., 2014; Hartmann et al., 2015; Cooper et al., 2019).

One critique of the EEfRT task and some of its variations is that it does not vary reward and effort equally. In the version of the task that has been most frequently deployed, there are five reward manipulations and only two effort conditions (Figure 3A; Barch et al., 2014). This makes it difficult to interpret how reward and effort processing individually contribute to altered decision making in schizophrenia. Specifically, it is not clear if the deficits in evaluating reward value across different magnitudes also extend to effort. Future work might benefit from utilising task structures which parametrically and symmetrically co-vary reward and effort. Additionally, it would be important to define negative symptom severity using more modern instruments like the brief negative symptom questionnaire (BNSS) (Kirkpatrick et al., 2011). Consistent use of these measures across studies might improve interpretability of associations between negative symptom severity and behavioural parameters.

While the EEfRT task manipulated physical effort, other studies have investigated the effects of *cognitive* effort on decision making in schizophrenia (Gold et al., 2014; Reddy et al., 2015, 2018; Culbreth et al., 2016, 2020; Chang et al., 2020). These include paradigms where participants chose to perform either a cognitively simple operation for a fixed-low reward, or a more demanding one for variable high rewards (Reddy et al., 2015, 2018). In one example, the two choices were represented by two decks of cards (Figure 3B; Reddy et al., 2018). In the “difficult” deck asked participants were asked to alternate between one of two mental operations (e.g., is the displayed number odd? Or is this number greater than five?), whereas the “easy” deck repeated a single operation across all trials (e.g., is this number greater than five?) (Reddy et al., 2018). Schizophrenia patients accepted far fewer offers in the high effort-high reward condition when compared to controls. However, there was no direct relationship between offers accepted and negative symptoms, although including mediating factors (e.g., dysfunctional beliefs such as: “Why bother, I’ll just fail again”) in the analyses yielded an indirect significant association (Reddy et al., 2018).

Another paradigm that has been used to examine cognitive effort is the “N-back” working memory task, where participants are asked if a displayed letter in a rapid, serial sequence is identical to the one presented “N” steps previously (Figure 3C; Westbrook et al., 2013). For example, the “easy” 1-back condition involves comparing each letter to the preceding one in the sequence and reporting if it is the same or different. On the other hand, the more challenging 4-back condition requires a comparison of each letter with the one shown four steps previously. Typically, participants were given a choice between a 1-back trial at low reward ($1) or a 2–4 back trial for high reward ($2) (Culbreth et al., 2016, 2020; Chang et al., 2020). Critically, the value of the low-reward condition was adjusted based on the previous trial’s response. For example, if the low-reward trial was rejected, in subsequent trials its value was increased stepwise up to an “*indifference point*” where participants were equally likely to choose either offer. The indifference point is a measure of how effort “discounts” reward across trial types, and was used to compute a per condition subjective value as well as an overall summary measure of value known as the area under the curve (AUC).

Schizophrenia patients discounted rewards more steeply than controls as a function of cognitive effort on the N-back task (Culbreth et al., 2016, 2020; Chang et al., 2020). This effect, as measured by the subjective value (Chang et al., 2020; Culbreth et al., 2020) or AUC (Culbreth et al., 2016), was specifically associated with negative symptom severity (Culbreth et al., 2016, 2020; Chang et al., 2020). However, the nature of these associations is not very clear. For example, in a recent study by Chang et al. (2020) the relationship between behaviour and negative symptoms varied depending on the outcome measure of interest. After taking the sum of anhedonia, asociality, and avolition scores on the BNSS questionnaire, schizophrenia patients were split down the median into amotivation^+^ and amotivation^–^ sub-groups. When comparing the proportion of high reward-high effort responses, both amotivation^+^ and amotivation^–^ sub-groups accepted fewer offers compared to healthy volunteers. On the other hand, a comparison of subjective value between groups revealed a significant decrease in this parameter the amotivation^+^ sub-group compared to controls, but there was no difference between the amotivation^+^ and amotivation^–^ patients. Finally, there was no association between the AUC measure and negative symptoms in this particular study, which contrasts with findings by other groups using very similar paradigms (Culbreth et al., 2016).

Taken together, these findings suggest that schizophrenia patients are impaired in the “option selection” process of motivated behaviour (Figures 3A–C). Specifically, they forego high reward opportunities when they are coupled with high effort costs. This effect is present regardless of whether the cost is physical (EEfRT) or cognitive (N-back). Investigations utilising the EEfRT task suggest these deficits might arise due to impairments in constructing reward value (Gold et al., 2013; Barch et al., 2014; Reddy et al., 2015; Treadway et al., 2015) while N-back experiments support an additional role for cognitive effort avoidance (Culbreth et al., 2016, 2020; Chang et al., 2020). Both behavioural deficits are associated with negative symptom severity, however, these relationships may be more nuanced than initially proposed. If the value of reward is discounted more easily in patients with negative symptoms, is this because they cannot experience pleasure?

### Interaction With Behavioural Goals: “Liking” but Not “Wanting”

It might be argued that schizophrenia patients are less motivated by reward because they experience less pleasure. However, the evidence points to the contrary (Berenbaum and Oltmanns, 1992; Horan et al., 2006; Burbridge and Barch, 2007; Herbener et al., 2007; Barch et al., 2016). In fact, schizophrenia patients frequently report similar levels of pleasure to healthy volunteers in response to positive experiences like consuming a sugary drink or watching a funny video clip (Berenbaum and Oltmanns, 1992; Horan et al., 2006). This phenomenon of preserved hedonic responses has been replicated throughout the literature, and is summarised in a meta-analysis by Cohen and Minor (2010). So why do patients continue to display poor motivation for rewards while enjoying pleasurable activities? One view is that it is not the hedonic response *per se* that is affected in schizophrenia, but the ability to anticipate future pleasure from self-guided action. In other words patients may not “want” to engage in pleasurable activities despite reporting that they “like” those activities (Gard et al., 2007; Herbener et al., 2007; Edwards et al., 2015; Moran and Kring, 2018).

Early research supporting this proposal involved serial collection of self-ratings from schizophrenia patients and healthy individuals over the course of a week (Gard et al., 2007). Participants reported how much pleasure they derived from current and future activities (e.g., eating dinner). Despite showing similar pleasure ratings while engaged in current activities, they reported significantly less anticipatory pleasure for future actions. Forthcoming, but not current, activity ratings were associated with familial and social functional outcome measures. Other paradigms relied on the presentation of serial images with positive (cupcake), neutral (chair), or negative (snake) associations (Herbener et al., 2007; Edwards et al., 2015; Moran and Kring, 2018).

Subtle methodological variations permitted separate measurements of both consummatory (i.e., current) and anticipatory (i.e., future) pleasure assessments. For example, in one setting a preceding symbol denoted the image category (e.g., +sign before positive image) and ratings were collected at this stage as well as during image presentation (Edwards et al., 2015). In another, images rated as highly pleasant or unpleasant at presentation were then associated with a neutral cue and subsequent ratings were made in response to the neutral cue presentation (Moran and Kring, 2018). In both studies, patients reported similar levels of consummatory but reduced anticipatory pleasure (Edwards et al., 2015; Moran and Kring, 2018). Further, they not only undervalued the pleasantness of future reward (Moran and Kring, 2018), but rated unpleasant images less severely in comparison to healthy participants (Edwards et al., 2015; Moran and Kring, 2018). This dampening of anticipatory pleasure was correlated with negative symptom severity (Moran and Kring, 2018).

Taken together, this evidence suggests that schizophrenia patients are less able to represent reward value when considering *future* actions–the “wanting” or instrumental aspects of motivated behaviour. Moreover, this is both associated with functional outcomes, as well as negative symptom severity. So why does a patient with negative symptoms lose the ability to represent future reward appropriately? Do they suffer from a general inability to learn about reward? Or might there be specific learning deficits that give rise to this?

### Reinforcement Learning of Reward Value

Several investigations have employed reinforcement learning paradigms to further explore the hypothesis that the representation of reward value is impaired in schizophrenia (Figure 3D; Gold et al., 2008, 2012; Strauss et al., 2011; Yilmaz et al., 2012; Moran et al., 2017; Hernaus et al., 2018; Culbreth et al., 2020). In one experiment conducted by Gold et al. (2012), participants were presented with image pairs of different landscapes and asked to choose the “correct” option. In half the trials, choosing the correct answer resulted in monetary gain (vs. no gain if incorrect), and in the other half it prevented monetary loss (vs. a loss if incorrect). Correct responses were reinforced at varying probabilities for each pair (i.e., they were rewarded on 90 or 80% of trials). After learning all possible associations, participants were asked to choose the best option between novel combinations of the original images in a “transfer” phase (Gold et al., 2012). Investigators were particularly interested in comparing the most frequently rewarded option (FR) with the most frequently loss avoidant one (FLA) (Figure 3D).

In the learning phase, schizophrenia patients with high negative symptom severity demonstrated impaired learning in the conditions rewarded with high probability (90%) when compared to controls. However, loss-avoidance learning was intact, suggesting that reward-based learning was specifically affected. When faced with a direct comparison between FR and FLA choices, healthy participants selected the FR option significantly more often than patients with high negative symptoms. In other words, healthy participants and patients without negative symptoms incorporated the expected value of responses in their decisions more frequently than patients with high negative symptoms.

In computational terms, updating decisions based on future expected value was considered to be encoded in a “model-based” process termed Q-learning (Hernaus et al., 2018). One hypothesis is that patients with high negative symptom severity have lower Q-learning rates. Instead, they select options more frequently based on the accumulation of unexpected positive outcomes, or reward prediction errors, regardless of the future expected value. This process is encoded in a “model-free” process known as the actor-critic model (Hernaus et al., 2018). In this scenario, avoiding loss and gaining reward both classify as positive outcomes when compared to their paired alternatives (no gain and loss, respectively). As a result, when directly compared, they are selected indiscriminately despite only one of these outcomes (gaining reward) increasing expected value. In summary, this study proposed that reward processing deficits in schizophrenia are characterised by a reduction in Q-learning and an increase in actor-critic learning.

Subsequently, the same group proposed that Q-learning deficits should be most noticeable when comparing options with the largest (i.e., easiest) discriminations in expected value (Hernaus et al., 2018). To address this possibility, Hernaus et al. (2018) used a similar paradigm to compare responses to image pairs with three different probabilities (90:10, 75:25, and 60:40). As expected, schizophrenia patients accepted less offers than controls at the highest (90:10 and 75:25) probabilities, but not lower probabilities (60:40). Using a logistic regression, the authors were able to confirm that the group differences could be detected as a function of the expected value difference (Hernaus et al., 2018).

Next, they attempted to capture these findings using a computational model that incorporated model-based (Q-learning) and model free (actor-critic) learning. The hypothesis was that the expected value difference could be modelled using a mixing parameter (*m*) representing the extent of model-based/model-free learning employed by each subject. Further, this parameter should relate to both group differences and possibly negative symptom severity (Hernaus et al., 2018). At the group level, the investigators were able to demonstrate that the *m* parameter captured the difference in expected value and was lower in schizophrenia patients compared to healthy volunteers. However, there was no association between this parameter and negative symptoms severity. This finding that reward based learning is most impaired at the highest probabilities has been reported across different learning paradigms (Yilmaz et al., 2012; Chang et al., 2016; Moran et al., 2017), but not accounted for with a single model previously.

In view of these results, one surprising finding across studies is that schizophrenia patients demonstrate intact general learning ability (Gold et al., 2012; Moran et al., 2017; Hernaus et al., 2018; Culbreth et al., 2020). That is, they can learn about rewards in general. For example, schizophrenia patients display similar overall accuracy in choices to controls in the transfer phase (Moran et al., 2017; Hernaus et al., 2018; Culbreth et al., 2020) and accurately favour frequently rewarding options over frequent loss ones (Gold et al., 2012). When faced with two relatively positive choices, however, they are less able to determine which response leads the best outcomes.

To sum up, schizophrenia patients appear to suffer impairments in reward-based learning due to a specific deficit in representing expected value. This impairment is specific to reward learning and not loss avoidance (Gold et al., 2012). Nevertheless, there still seems to be a lack of clarity about how exactly these behavioural deficits specifically relate to negative symptoms severity. One possible explanation is that the behavioural deficits related to negative symptoms are mediated by factors including cognitive impairment, dopaminergic treatment, or dysfunctional attitudes. We explore these possibilities briefly in the following section.

### Cognitive Dysfunction and Reward Processing

Cognitive dysfunction is a well-documented core phenomenon in schizophrenia (Heinrichs and Zakzanis, 1998; Kahn and Keefe, 2013; Schaefer et al., 2013), but the association with negative symptom questionnaires is complex (Harvey et al., 2006). While questionnaire analyses reveal correlations between negative symptoms and cognitive impairment (O’Leary et al., 2000; Good et al., 2004), these associations are often modest and weakened when adjusting for confounding factors such as functional outcomes (Heydebrand et al., 2004; Bowie et al., 2006; Couture et al., 2011). In the case of EBDM, impaired cognitive function has been shown to be associated with altered decision making for rewards (Heerey et al., 2007, 2008). Notably, a recent re-analysis of several studies using the EEfRT task demonstrated that schizophrenia patients with difficulties in constructing value representations were characterised by greater impairments across measures of cognitive function (Cooper et al., 2019). Hence, reward sensitivity is not only altered by negative symptoms but also by cognitive impairment.

These findings suggest negative symptoms and cognitive dysfunction are, at the very least, highly interdependent and might operate in tandem to regulate motivated behaviour (Robison et al., 2020). A better characterisation of this interaction at the behavioural level would be imperative in uncovering the relationship between negative symptoms, cognitive impairment and EBDM. One emerging hypothesis suggests that altered behaviour associated with negative symptoms and cognitive impairment is mediated by dysfunctional attitudes and/or defeatist beliefs (Grant and Beck, 2009; Horan et al., 2010; Quinlan et al., 2014). So far, two investigations have additionally associated these beliefs to performance on EBDM tasks (Granholm et al., 2016; Reddy et al., 2018).

In one study, patients with high scores on self-defeatist beliefs demonstrated reduced pupillary dilation while recalling increasingly complex numeric sequences (Granholm et al., 2016). Additionally, negative symptom severity positively correlated with self-defeatist beliefs. More recently, Reddy et al. (2018) showed that schizophrenia patients accepted less offers at high reward-high cognitive effort compared to healthy volunteers. While there was no direct association between responses and negative symptoms, when the interaction term between negative symptoms and self-defeatist beliefs was included as a covariate, there was a significant positive association between the number of offers accepted and this interaction term (Reddy et al., 2018). Additionally, cognitive impairment was the strongest predictor of task performance across analyses, but the authors did not explore the interactions between cognitive function, negative symptoms, and dysfunctional beliefs. These preliminary findings suggest that mediating factors like cognition and/or dysfunctional beliefs may play a role in linking EBDM deficits and negative symptoms in schizophrenia. Hypothesis driven mediation analyses in future behavioural work may further clarify these complex associations.

### Dopamine and Effort-Based Decision Making

Regulation of the neurotransmitter dopamine is heavily implicated both in EBDM and the pathophysiological changes in schizophrenia (Davis et al., 1991; Salamone et al., 2007; Maia and Frank, 2017). Additionally, most antipsychotic medications used in schizophrenia target dopaminergic D2 receptors (Miyamoto et al., 2005). Several studies have found no associations between antipsychotic dose equivalents and negative symptoms (Gold et al., 2013; Chang et al., 2020). Crucially, they also failed to find a relationship between EBDM deficits and negative symptom severity (Fervaha et al., 2013; Gold et al., 2013; Barch et al., 2014; Chang et al., 2020).

One interpretation of this lack of association is that most investigations do not dissociate between antipsychotic medication based on D2 receptor affinity (Gold et al., 2015). Specifically, Gold et al. (2015) showed in a small sample that patients on first generation antipsychotics were significantly less likely to expend effort for rewards in comparison to patients on second generation medications like clozapine. However, this was based on a small sample size (16 cases on clozapine) and a more sizeable replication of this work is necessary.

A recent analysis of 196 patients demonstrated an inverse association between negative symptom severity and medication dose that was specific for atypical antipsychotic therapy, but this study did not investigate effort-based decisions (Fujimaki et al., 2018). There would be considerable potential value in a conducting well-powered EBDM tasks in patients on different antipsychotic medications. Demonstrating distinct behavioural and questionnaire responses on first vs. second generation anti-dopaminergic drugs might influence both current clinical practice as well as future therapeutic development.

### Neural Correlates of Reward Processing in Schizophrenia

**FIGURE 4:**
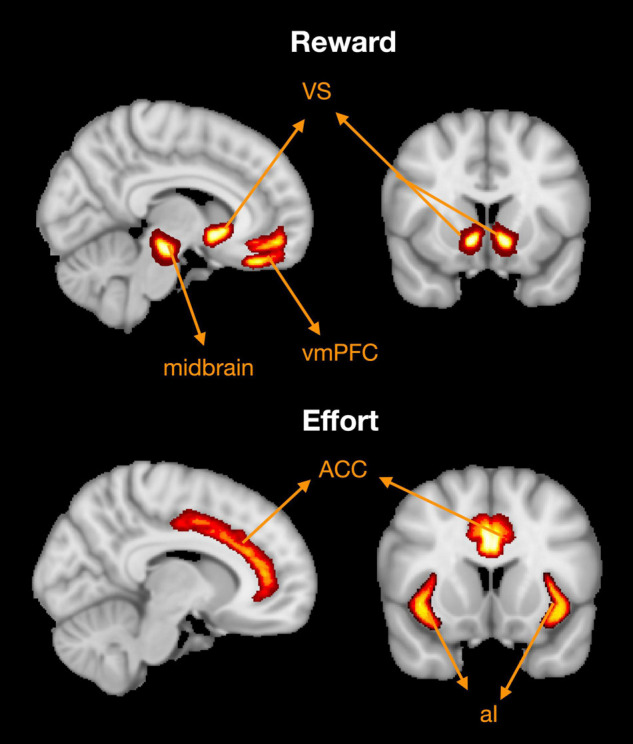
Brain regions associated with reward and effort processing. Based on a meta-analysis of functional MRI studies (Pessiglione et al., 2018). Reward processing associated with activation in the ventromedial pre-frontal cortex (vmPFC), ventral striatum (VS), and ventral tegmental area within the midbrain. Effort processing clusters are principally found in the dorsal anterior cingulate cortex (ACC) and anterior insula (AI). Adapted from Figure 5 in Pessiglione et al. (2018).

The results of neuroimaging studies in healthy people have revealed that reward related processing occurs in distinct areas of the brain (Figure 4; Pessiglione et al., 2018). Typically, these are regions in the medial frontal cortex and basal ganglia: the orbitofrontal cortex (OFC) sometimes referred to as the ventromedial pre-frontal cortex (vmPFC), anterior cingulate cortex (ACC), ventral striatum (VS) including the nucleus accumbens, and the ventral tegmental area (VTA) of the midbrain (Croxson et al., 2009; Bartra et al., 2013; Bonnelle et al., 2016). Tracer studies in animals and tractography analyses in humans have revealed that these areas are interconnected in an extensive network (Haber and Knutson, 2010). Functional neuroimaging studies demonstrate that both the OFC and VS are activated in anticipation of rewards (Croxson et al., 2009; Kroemer et al., 2014), while the ACC encodes subjective value by integrating reward and effort signals (Croxson et al., 2009; Bonnelle et al., 2016). Lesions studies offer additional support for these findings. Specifically, OFC and VS lesions are associated with altered sensitivity to rewards as measured by pupillary responses (Manohar and Husain, 2016). Recently, Manohar and Husain (2016) showed that patients with vmPFC lesion completing a gambling task were less reliant on the expected value of rejected options when compared to controls. Additionally, lesions within the ACC are associated with severe forms of apathy known as akinetic mutism (Barris and Schuman, 1953; Németh et al., 1988), whereas lesions in the basal ganglia, and thalamus can also lead to significant decreases in motivation, although this may improve with dopaminergic therapy (Adam et al., 2013; Blundo and Gerace, 2015). Preclinical studies lend further support to the role of these brain regions in facilitating motivated behaviour, namely through dopaminergic transmission (reviewed in Floresco et al., 2008; Salamone et al., 2018). In food deprived rodents, dopamine depletion within the nucleus accumbens leads to significant reductions in effort exertion during feeding behaviour (Salamone, 1988; Salamone et al., 1991; Koch et al., 2000). Together, this evidence implicates a distinct neural network in the regulation of motivated behaviour, possibly through dopaminergic transmission.

Might these brain regions be disrupted in schizophrenia, and what is their association with negative symptom severity? Early evidence from Functional MRI studies suggests both schizophrenia and negative symptom severity are associated with impairments within the fronto-striatal network (Walter et al., 2009; Dowd and Barch, 2012; Arrondo et al., 2015; Subramaniam et al., 2015; Moran et al., 2019; Shukla et al., 2019; Xu et al., 2019). Neural activation, inferred from blood oxygen level dependent (BOLD) signals, was compared between patients and healthy controls while performing various reinforcement learning paradigms (Walter et al., 2009; Dowd and Barch, 2012; Arrondo et al., 2015; Subramaniam et al., 2015). One example involved responding with a key press to either a positive cue (e.g., upwards arrow) or neutral cue (e.g., horizontal arrow). Positive cues were followed by one of two monetary rewards (a pound vs. a penny), whereas neutral cues preceded one of two colours (yellow vs. brown circle). When anticipating rewards, schizophrenia patients showed less activation in several brain regions in comparison to healthy participants (Walter et al., 2009; Dowd and Barch, 2012; Arrondo et al., 2015; Moran et al., 2019). These included the VS (Walter et al., 2009; Arrondo et al., 2015), ACC (Walter et al., 2009; Arrondo et al., 2015; Moran et al., 2019), and OFC (Arrondo et al., 2015). Blood oxygen level dependent signal strength in these regions was found to be negatively associated with MAP symptoms in patients (Moran et al., 2019), although this finding is not consistent (Dowd and Barch, 2012; Arrondo et al., 2015). For example, two earlier experiments demonstrated that reduced VS activation during reward anticipation was only associated with anhedonia, and not other components of the negative syndrome (Dowd and Barch, 2012; Arrondo et al., 2015). Functional connectivity has also been assessed in schizophrenia (Shukla et al., 2019; Xu et al., 2019). This involves voxel-wise correlation of BOLD signal time series between spatially distributed brain areas (Shukla et al., 2019; Xu et al., 2019). The assumption is that regions that show high similarity in BOLD signal are likely to be functionally connected (Woodward and Cascio, 2015). Compared to 139 healthy volunteers, 95 schizophrenia patients showed significant reductions in functional connectivity within the striatum-OFC and striatum-ACC (Shukla et al., 2019). Additionally, the extent of the impairment within the striatum-OFC was dependent on negative symptom severity. This finding was replicated in a cohort study of 84 schizophrenia patients, where VTA-OFC connectivity was negatively associated with social amotivation (Xu et al., 2019). Together, this evidence suggests that negative symptoms in schizophrenia patients are associated with reduced functional activation during reward anticipation as well as altered functional connectivity within the fronto-striatal network.

Clinical apathy in neurological conditions like Parkinson’s disease (Baggio et al., 2015) and cerebrovascular small vessel disease (Hollocks et al., 2015; Saleh et al., 2021) is also associated with structural and functional deficits within the medial forebrain regions and the basal ganglia, including the VS (Hollocks et al., 2015; Le Heron et al., 2018a; Saleh et al., 2021). This suggests that some negative symptoms, like apathy/motivation may have similar underlying neural origins, across different diseases (Husain and Roiser, 2018). Such a view has been recently proposed by Strauss and Cohen (2017) in a transdiagnostic view of negative symptoms.

## Summary and Conclusion

Negative symptoms are an important feature of schizophrenia. Several lines of evidence suggest that they have a deleterious impact on functional outcomes, so finding treatments that could ameliorate them are considered an important goal of current research. Our understanding of the “negative syndrome” might be advanced by using well-designed EBDM tasks that probe the different phases of behaviour (Figure 2). To date, these paradigms reveal consistent deficits in motivated decision making when evaluating rewards against physical (EEfRT) or cognitive (N-back) effort (Gold et al., 2013; Barch et al., 2014; Chang et al., 2020; Culbreth et al., 2020). This may be due to one or a combination of reduced reward sensitivity and increased effort avoidance. Deeper exploration of schizophrenia patients’ hedonic responses demonstrates that negative symptoms do not reduce the experience of “liking” pleasure (Barch et al., 2016). Instead, they anticipate less enjoyment from future activities, and might therefore “want” to do less than healthy people.

Assessments using reinforcement learning paradigms suggest that reward processing deficits arise in patients because they put less emphasis on the expected value of decisions (Hernaus et al., 2018). Instead, they are guided by the basic stimulus-response relationships between action and outcome. Computational models which attempt to frame behavioural deficits this way are very appealing, but while they capture overall behaviour they do not currently consistently relate it to negative symptoms (Hernaus et al., 2018). Perhaps such models may overfit the data, and subsequently affect associations with questionnaires. Alternatively, it might be that conventional negative symptom questionnaires do not provide the most robust link to the behavioural observations of interest. One factor complicating interpretation is that different studies are inconsistent in which questionnaires they use.

Another is that the relationship between negative symptoms and reward representation might be mediated by other factors. There is increasing interest in how cognitive impairment, or accompanying dysfunctional beliefs, might bridge the association between negative symptoms and altered effort-based decisions (Reddy et al., 2018; Cooper et al., 2019; Robison et al., 2020). This area may prove valuable in explaining some of the inconsistencies between studies when relating deficits in reward based learning and option selection to negative symptoms. Similarly, there is a gap in the current understanding of how treatment with different dopamine receptor antagonists differentially impacts negative symptoms and EBDM.

Negative symptoms are associated with specific changes within a fronto-striatal network that is closely associated with EBDM (Moran et al., 2019; Shukla et al., 2019; Xu et al., 2019). This is particularly interesting as this network is also associated with clinical apathy in neurological diseases (Hollocks et al., 2015; Saleh et al., 2021). Exploring the neural and behavioural differences between negative symptoms across diseases is an exciting future area of research. An important factor in pursuing transdiagnostic enquiries of negative symptoms is to account for variations in terminology across clinical specialties. For example, negative symptoms include avolition, anhedonia, and asociality under a single rubric (MAP). On the other hand, neurologists and mood disorder specialists often refer to clinical apathy as an independent syndrome which may overlap with anhedonia (Husain and Roiser, 2018). Further, clinical apathy as defined in this manner contains several symptom dimensions (e.g., behavioural, social, and emotional) which overlap with MAP symptoms (Marin et al., 1991; Kirkpatrick et al., 2006; Sockeel et al., 2006; Ang et al., 2017). Behaviourally, apathetic patients with cerebrovascular (Le Heron et al., 2018a; Saleh et al., 2021), and Parkinson’s disease (Le Heron et al., 2018b) display reward processing deficits when performing an EBDM task. Specifically, they accept less monetary offers at *low* reward compared to their motivated counterparts (Le Heron et al., 2018a,b; Saleh et al., 2021). This is a slightly different behavioural pattern to that demonstrated in schizophrenia patients with negative symptoms, where less offers are accepted at the *highest* reward levels (Gold et al., 2013, 2015). These subtle differences imply that while both syndromes alter reward processing, their behavioural phenotypes are not identical. Differences may be due to diverging pathological processes at the neural level, specific co-morbidities (e.g., cognitive impairment), or pharmacological interactions (e.g., with anti-dopaminergic medication). Transdiagnostic investigations across motivation disorders will ensure that these differences are better defined and accounted for. Additionally, using questionnaires designed specifically for transdiagnostic purposes can be valuable when comparing findings across patient groups.

One limitation of the conceptual framework discussed here is that it does not address components and contributors of negative symptoms outside of EBDM. Specifically, while we explore negative symptoms at the individual level we do not discuss the environmental contributions to negative symptom severity, which can be significant (van Os et al., 2002; Oshima et al., 2005; Van Os et al., 2010). A recent review poses a theoretical framework of how this can be integrated within individual factors (Strauss, 2021). While our current concepts of negative symptoms remains relatively modest, future approaches might explore the interactions between environmental factors and EBDM at the individual level. A related consideration is whether the expressive symptoms (alogia/blunted affect) of schizophrenia can be similarly quantified using behavioural tasks. While effort-based decisions seem to be more influenced by motivational deficits (anhedonia/avolition/asociality), there is an increasing body of literature using different approaches to quantify both emotional (Trémeau, 2006; Carter et al., 2009; Prochwicz and Rózycka, 2012) and verbal (Cohen et al., 2014) deficits in schizophrenia. These include facial recognition tasks (Carter et al., 2009), as well as speech analysis techniques (Cohen et al., 2020), both of which may offer valuable contributions to our future understanding of negative symptoms (Jeganathan and Breakspear, 2021).

In summary, motivational deficits are an important facet of schizophrenia and its underlying mechanisms has the potential to be advanced through well-designed behavioural and neuroimaging research. A better understanding of how amotivation alters reward-processing and which brain regions are implicated can provide the foundations for future treatment strategies. The transdiagnostic nature of these deficits poses the distinct possibility that future treatments for motivation deficits in schizophrenia might benefit patients with other brain disorders.

## Author Contributions

YS conducted the literature review, critically analysed the literature, and drafted the manuscript and figures. IJ-B contributed to the reading and writing of the manuscript. EF-E performed the critical revision and approval of the current version. MH performed the overall design and planning of review, critical revision, and approval of the current version. All authors contributed to the article and approved the submitted version.

## Conflict of Interest

The authors declare that the research was conducted in the absence of any commercial or financial relationships that could be construed as a potential conflict of interest.

## Publisher’s Note

All claims expressed in this article are solely those of the authors and do not necessarily represent those of their affiliated organizations, or those of the publisher, the editors and the reviewers. Any product that may be evaluated in this article, or claim that may be made by its manufacturer, is not guaranteed or endorsed by the publisher.

## Abbreviations

NIMH: National Institute of Mental Health
EXP: diminished expression factor within the negative syndrome
MAP: motivation and pleasure factor within the negative syndrome
PANSS: positive and negative syndrome scale
SANS: scale for the assessment of negative symptoms
EBDM: effort based decision making
EEfRT: effort expenditure for rewards task
BNSS: brief negative symptom questionnaire
AUC: area under the curve
FR: frequently rewarded
FLA: frequently loss avoidant
OFC/vmPFC: orbitofrontal cortex/ventromedial pre-frontal cortex
ACC: anterior cingulate cortex
VS: ventral striatum
VTA: ventral tegmental area
MRI: magnetic resonance imaging
BOLD: blood level oxygen dependent.
